# Thyroid Stimulating Hormone circadian variations in paranoid schizophrenic psychosis between acute and stable phases. A comparative study.

**DOI:** 10.1192/j.eurpsy.2023.445

**Published:** 2023-07-19

**Authors:** A. Marcos-Rodrigo, A. L. Morera-Fumero, P. Abreu-Gonzalez, E. Diaz-Mesa, L. Fernandez-Lopez, J. J. Tascon-Cervera

**Affiliations:** ^1^Servicio de Psiquiatria, Hospital Universitario Nuestra Señora de Candelaria, Santa Cruz de Tenerife; ^2^Departamento de Medicina Interna, Dermatología y Psiquiatría, Facultad de Ciencias de la Salud; ^3^Ciencias Medicas Basicas: Unidad de Fisiologia, Facultad de Ciencias de la Salud; ^4^Servicio de Psiquiatria. Hospital Universitario de Canarias. Departamento de Medicina Interna, Dermatologia y Psiquiatria, Universidad de La Laguna; ^5^Servicio de Psiquiatria, Hospital Universitario de Canarias, San Cristobal de La Laguna, Spain

## Abstract

**Introduction:**

Day-night changes in several molecules are studied as biomarkers of circadian rhytms (Morera-Fumero, A. L. *et al.* Progress in Neuro-Psychopharmacology and Biological Psychiatry 2017; 75 207-212). Circadian rhythmicity of the pituitary-thyroid axis has been proven in healthy individuals, with a Thyroid Stimulating Hormone (TSH) peak in serum around midnight and peaks during day hours (Bellastella, G. *et al.* Life 2021; 11(5), 426). A recent meta-analysis has reported differences in serum TSH levels between first-episode psychosis and multiple-episode schizophrenia (Misiak, B. *et al.* Progress in Neuro-Psychopharmacology and Biological Psychiatry 2021; 111, 110402). However, studies assessing quantitative circadian variations on TSH serum in schizophrenic patients are scant.

**Objectives:**

Comparing serum TSH levels at two different times of the day (12:00 and 24:00 hours) and the differences between the acute (hospital admission) and recovered phase (hospital discharge) of the disease.

**Methods:**

Fourteen male patients (age 26,8±9,3 years) with the diagnosis of paranoid schizophrenia psychosis according to the DSM-IV partake in the study. Patients were admitted to the University Hospital of the Canary Islands psychiatric room because of acute relapse. Blood samples were taken in the first 24 h of admission and at 24 h. before discharge. All patients gave written consent to participate in the research study. Serum TSH was determined by ELISA methods. Paired sample t-tests were performed between TSH serum levels at admission and discharge at 12:00 and 24:00 hours. Statistical analyses were performed using IBM® SPSS® Statistics 25 software for MAC (IBM Corporation 1989, 2017).

**Results:**

There were statistical differences between the 12:00 h and the 24:00 h of the TSH serum levels at admission (12:00: 145,856±156,961vs. 00:00: 192,006± 122,757, p = 0.04); TSH discharge, (12:00: 134,483±72,882vs 00:00: 244,214±148,697, p = 0.002). There were no statistical differences between the 12:00 TSH levels at admission and discharge (145,856±156,961 vs. 134,483± 72,882, p = 0.66). The 24:00 h comparison of TSH levels neither elicited significant results (admission: 192,006±122,757 vs. discharge: 244,214± 148,697, p = 0.15).

**Conclusions:**

Schizophrenic patients undergo TSH serum changes in a circadian pattern during the acute and stable phases of the disease; nevertheless, they experience smaller deviations during the acute phase. Higher levels of TSH were observed around midnight, as it happens in healthy individuals, with higher peaks during the stable phase compared to the acute one.

**Disclosure of Interest:**

None Declared

